# Effect of Prophylactic Antibiotics on K-Wire-Associated Infection: A Retrospective Cohort Study

**DOI:** 10.7759/cureus.101334

**Published:** 2026-01-12

**Authors:** Mohammad A Tahir, Haroon Z Malik, Sohail Nisar

**Affiliations:** 1 Department of Emergency Medicine, Hull Royal Infirmary, Hull, GBR; 2 Department of Emergency Medicine, Whiston Hospital, Prescot, GBR; 3 Department of Orthopaedics, York Hospital, York, GBR

**Keywords:** antibiotic stewardship, fracture of distal radius, kirschner wire (k-wire), prophylactic antibiotics, supracondylar humeral fracture

## Abstract

Introduction

Orthopaedic surgeons frequently manipulate and fix fractured bones into place using metal pins known as Kirschner wires (K-wires). The percutaneous placement of these wires makes them susceptible to infection at the pin site. Some surgeons choose to administer antibiotics prophylactically to prevent pin-site infection, while others do not; this variability is due to a lack of conclusive evidence in support of either practice. This study aims to investigate whether prophylactic antibiotics reduce the incidence of pin site infection by comparing those who received prophylaxis with those who did not.

Methods

A two-centre retrospective cohort study was performed. Records were reviewed for patients who underwent K-wire fixation of either supracondylar humerus or distal radius fractures at Leeds General Infirmary or Bradford Royal Infirmary between 2007 and 2021. Data was collected on antibiotic administration, pin-site infections, fracture location, age, sex, and comorbidities.

Results

A total of 264 patients met the eligibility criteria and had complete patient records. The overall incidence of K-wire-associated infection was 3.4%. In the group that received antibiotic prophylaxis, there was a higher incidence of infection (3.9% vs 2.4%); however, this result was not significant (OR 1.6, 95% CI 0.33-7.87, p=0.73).

Conclusions

This study detected no reduction in infection rates when using antibiotic prophylaxis; however, the results did not reach significance. The main study limitation was a small sample size. Due to infection rates being low, the study was underpowered to detect a small effect size. It is recommended that future randomised controlled trials and meta-analyses be carried out to provide significant conclusions and inform future practice.

## Introduction

Kirschner wires (K-wires) are smooth, rigid wires used as a simple, effective and inexpensive method of bone fixation [1]. These temporary wires are usually removed after four to six weeks once the fracture is stable. K-wires may be buried subcutaneously or left with an exposed end, which is then clipped and bent; both placements are equally effective at fracture fixation [2,3]. The overall rate of K-wire-associated infection is reported to be between 1-7% [4-7]; however, exposed K-wires have been demonstrated to have an increased infection risk due to the break in the skin barrier, with a reported relative risk of 2.04 [8,9].

Prophylactic antibiotic administration is one approach to reducing the risk of K-wire-associated infection. The National Institute for Health and Care Excellence (NICE) guideline NG125 on surgical site infections recommends antibiotic prophylaxis before clean surgery involving implant or prosthesis placement [10]. However, due to a lack of guidance specific to K-wires, there is variability in clinical practice. In a survey of 303 British Orthopedic surgeons, 50% responded that they routinely administer antibiotics, 28% said they never do, and the remaining 22% consider them on a case-by-case basis [11]. Despite low overall infection rates, the benefit of prophylactic antibiotics in reducing K-wire-associated infection is a pertinent clinical issue. Inappropriate antibiotic use increases cost, promotes bacterial resistance and exposes patients to the risk of adverse drug effects. Conversely, demonstration of a modest benefit from antibiotics could still justify their prescription, as small reductions in infection rates could translate to fewer clinic visits and lower numbers of serious complications such as osteomyelitis. 

The objective of this study is to determine the effect of prophylactic antibiotics on the incidence of K-wire-associated infection in distal radius fractures (DRF) and supracondylar humerus (SCHF) fractures. Our hypothesis is that the use of antibiotics will not reduce the incidence of infection.

## Materials and methods

A two-centre retrospective observational cohort study was performed on patients treated at Leeds General Infirmary between 2007 and 2017 and Bradford Royal Infirmary between 2019 and May 2021. Inclusion criteria were patients who underwent K-wire fixation of DRF or SCHF and were followed up until the time of pin removal. These fracture types were selected as they comprise the majority of procedures utilising K-wires at these two centres and also represent a relatively homogeneous cohort, both being long-bone upper-limb fractures managed with a similar percutaneous K-wire fixation technique. Patients with polytrauma, open fractures or previous same-site trauma were excluded.

K-wire fixation was performed in the operating theatre using steel wires with sterile technique and draping. Following insertion, the wires were clipped, bent and left unburied. Non-adhesive paraffin gauze (Jelonet; Smith+Nephew, Hertfordshire, UK) dressings were then applied.

A total of 556 patients were identified, and their electronic patient records were reviewed. These were composed of some entirely electronic notes and some that were paper notes, which had been scanned onto the patient record. 292 (52.5%) of these patients were either lost to follow-up or had missing anaesthetic or operative notes due to not being scanned. The missing data was due entirely to administrative challenges relating to scanning and uploading paper records and not due to any clinical or patient-related factors. For these patients, their antibiotic status could not be determined, and so they were excluded. The final sample comprised 264 patients.

The study exposure was the administration of antibiotic prophylaxis. The choice of antibiotic was determined by the surgeon's preference; these included IV teicoplanin 400mg + gentamicin 5mg/kg (37 patients), IV flucloxacillin 1g (78 patients), IV co-amoxiclav 1.2g (49 patients), or IV cefuroxime 1.5g (18 patients). All were administered as a single intraoperative dose. The primary outcome was the development of K-wire-associated pin-site infection, defined as any patient prescribed antibiotics due to signs of infection such as purulent discharge, erythema, warmth or swelling at the K-wire site. Demographic data was collected on age, sex, diabetes and smoking status. Clinical variables collected included fracture location and operative time.

Patients were separated into two groups, depending on whether they had received antibiotic prophylaxis or not. Analysis between groups was performed using an independent Welch’s two-tailed T-test for mean age and operative time. The categorical variables of infection, fracture location, sex, smoking and diabetes were analysed using the chi-squared test, except where counts were less than five, and so the Fisher’s Exact test was used. A further exploratory analysis was carried out between the infected and the non-infected groups, utilising the same statistical approach. A p-value <0.05 was considered significant. Statistical analysis was performed using Jamovi version 2.7.11 (www.jamovi.org). To contextualise the study's ability to detect modest effect sizes, a retrospective sample size calculation was performed. Assuming a power of 80% and a two-sided α of 0.05, to detect a reduction in infection rate from 3.5% to 1% would require 551 patients in each group.

The study protocol was reviewed and approved by the University of Leeds, School of Medicine research team, and it was deemed exempt from ethical approval.

## Results

A total of 264 patients were included in this study. The mean age was 26.3 ± 20.0 years, 145 (54.9%) were male, and 119 (45.1%) were female. 161 (61%) were treated for a DRF and 103 (39%) for a SCHF. Demographics and baseline characteristics are shown in Table 1. 182 (68.9%) of the patients received antibiotics, while 82 (31.1%) did not. A comparison of demographic and baseline characteristics between groups is shown in Table 2.

**Table 1:** Baseline characteristics of included patients DRF: distal radial fracture; SCHF: supracondylar humerus fracture

Characteristic	Value
Age, mean ± SD, years	26.3 ± 20.0
Sex, n (%)	
Male	145 (54.9)
Female	119 (45.1)
Fracture Location, n (%)	
DRF	161 (61.0)
SCHF	103 (39.0)
Smokers, n (%)	5 (1.89)
Diabetes, n (%)	3 (1.14)

**Table 2:** Comparison of patient characteristics between cohorts Statistical testing was performed with a Welch's t-test for age and operative time, a chi-square test for sex and fracture type, and Fisher's exact test for diabetes and smoking. DRF: distal radial fracture; SCHF: supracondylar humerus fracture

Variable	Antibiotic (n=182)	No antibiotic (n=82)	Test statistic value (df)	p-value
Age, mean ± SD, years	26.6 ± 20.7	25.5 ± 18.2	0.44 (175)	0.66
Sex, n (%)			0.08 (1)	0.78
Male	101 (55.5)	44 (53.7)		
Female	81 (44.5)	38 (46.3)		
Fracture type, n (%)			1.19 (1)	0.28
DRF	107 (58.8)	54 (65.9)		
SCHF	75 (41.2)	28 (34.1)		
Operative time, mean ± SD, min	59.4 ± 32.1	53.5 ± 24.8	1.52 (162)	0.13
Smokers, n (%)	3 (1.65)	2 (2.44)	N/A	0.65
Diabetes, n (%)	3 (1.65)	0 (0)	N/A	0.56

The overall incidence of infection in this study was 3.41%, with a total of nine infections. 7 (3.85%) of the patients in the antibiotic prophylaxis group developed infections compared to 2 (2.44%) of the patients in the no antibiotic group, as shown in Table 3. The difference in infection rates between cohorts was not statistically significant, p=0.73. In the secondary analysis between the infected and non-infected groups, there was no significant difference in any measured variable. The results of this analysis are shown in Table 4.

**Table 3:** Incidence of infection between groups Statistical testing was performed with a Fisher's exact test.

	Antibiotic (n=182)	No antibiotic (n=82)	Odds Ratio (95% Confidence Interval)	p-value
Infection, n (%)	7 (3.85)	2 (2.44)	1.60 (0.33 – 7.87)	0.73

**Table 4:** Secondary analysis between infected and non-infected patients Statistical testing was performed with a Welch's t-test for age and operative time, and Fisher's exact test for sex, fracture type, smoking and diabetes. DRF: distal radial fracture; SCHF: supracondylar humerus fracture

Variable	Infected (n=9)	Not infected (n=255)	Test statistic value (df)	p-value
Age, mean ± SD, years	31.7 ± 22.1	26.1 ± 19.8	0.75 (8.46)	0.48
Sex, n (%)			N/A	0.52
Male	6 (66.7)	139 (54.5)		
Female	3 (33.3)	116 (45.5)		
Fracture type, n (%)			N/A	0.49
DRF	7 (77.8)	154 (60.4)		
SCHF	2 (22.2)	101 (39.6)		
Operative time, mean ± SD, min	50.3 ± 16.1	58.0 ± 30.7	-1.33 (10.4)	0.21
Smokers, n (%)	0 (0)	5 (1.96)	N/A	1.00
Diabetes, n (%)	0 (0)	3 (1.18)	N/A	1.00

## Discussion

The overall incidence of K-wire-associated infection in this study was 3.41%, which is consistent with the range reported in previous studies of between 1-7% [4-7]. The infection rate was higher in the group of patients who received antibiotic prophylaxis (3.85% vs. 2.44%). This aligns with the findings of a recent randomised controlled trial [5]; however, in both cases, the result was not statistically significant. Our study, therefore, detected no significant effect of administering antibiotic prophylaxis to lower the risk of K-wire-associated infection.

This is contrasted by a single study that reported a higher incidence of K-wire infections in patients who didn’t receive antibiotic prophylaxis (6.5% vs 2.2%); consequently, the authors concluded that antibiotic use lowers infection risk and antibiotics should always be administered [12]. Notably, that study had a small sample size of only 77 patients and only three total infections. It also deviated from other studies in the duration of antibiotic administration, which began pre-operatively and continued 48 hours post-operatively, with an option to extend to five days post-operatively with oral antibiotics. The majority of literature, including our study, evaluates the effects of single doses pre-operatively or intra-operatively, with no significant effects on infectious outcomes found [13,14,15]. 

When comparing the patients that received antibiotic prophylaxis versus those that did not, there were no significant differences between them with regard to fracture type, age, smoking status or diabetic co-morbidity. No rationale behind the decision to prescribe prophylaxis was documented in the clinical notes. A recent survey of 302 British orthopaedic surgeons reiterated this lack of consensus on which patient or clinical characteristics may prompt antibiotics [11]. Among the 22% of respondents who prescribed antibiotics on a case-by-case basis, there were no standardised criteria between them that determined this choice.

In keeping with previous studies, for the patients who developed infection, there were no observed significant associations with age, sex, operative time, fracture location, smoking status or diabetes [7]. This should be interpreted cautiously in the context of a low number of infection events. Our study also only evaluated distal radius fractures and supracondylar humerus fractures; another study covering more fracture types demonstrated that fracture location can impact the incidence of K-wire infection, showing significantly higher infection rates for wires placed in the metacarpals over those in the distal radius [6,9].

A limitation of our study was that a large proportion of the originally identified patients were excluded due to missing data resulting from administrative challenges in scanning paper notes onto the electronic patient record. The risk of selection bias is likely low, as the missingness was due entirely to administrative reasons and not related to any clinical or patient-related factors. However, as it was not possible to analyse and compare the included and excluded patients for systematic differences, selection bias cannot be completely ruled out.

Another limitation of our study was sample size: due to a low incidence of infection in K-wire fixations, our study was underpowered to detect a small or modest effect size. This limitation is shared with all the other studies in this area that are also constrained by sample size [5-7, 12, 14, 15]. One meta-analysis evaluated four retrospective single-centre studies and one randomised controlled trial investigating the efficacy of antibiotic prophylaxis for K-wire-associated infection [13]; however, some of the patients included in this analysis underwent a semi-sterile fixation technique, which is not routinely practised in the UK. Our study provides a useful addition of data, which can be used to inform future meta-analyses on this topic.

## Conclusions

This study detected no benefit of antibiotic prophylaxis to reduce the incidence of K-wire-associated infection in DRF and SCHF. Further studies with larger sample sizes that are adequately powered are needed to detect a small or modest effect size.
